# Incorporating coronary artery calcium scoring in the prediction of obstructive coronary artery disease with myocardial ischemia: a study with sequential use of coronary computed tomography angiography and positron emission tomography imaging

**DOI:** 10.1007/s12350-022-03132-z

**Published:** 2022-11-15

**Authors:** Inge J. van den Hoogen, Xu Wang, Steele C. Butcher, Teemu Maaniitty, Jussi Schultz, Alexander R. van Rosendael, Antti Saraste, Juhani Knuuti, Jeroen J. Bax

**Affiliations:** 1 Department of Cardiology, Leiden University Medical Center, Albinusdreef 2, Postal zone 2300 RC, 2333 ZA Leiden, the Netherlands; 2 Department of Cardiology, Beijing Anzhen Hospital, Capital Medical University, Beijing Institute of Heart Lung and Blood Vessel Disease, Beijing, China; 3 Department of Cardiology, Royal Perth Hospital, Perth, WA Australia; 4 Turku PET Centre, Turku University Hospital and University of Turku, Turku, Finland; 5 Heart Center, Turku University Hospital and University of Turku, Turku, Finland

**Keywords:** Coronary artery calcium scoring, Coronary artery disease, Coronary computed tomography angiography, Myocardial ischemia, Myocardial perfusion imaging, Positron emission tomography

## Abstract

**Background:**

Additional strategies are needed to refine the referral for diagnostic testing of symptomatic patients with suspected coronary artery disease (CAD). We aimed to compare various models to predict hemodynamically obstructive CAD.

**Methods and results:**

Symptomatic patients with suspected CAD who underwent coronary artery calcium scoring (CACS) and sequential coronary computed tomography angiography (CCTA) and [^15^O]H_2_O positron emission tomography (PET) myocardial perfusion imaging were analyzed. Obstructive CAD was defined as a suspected coronary artery stenosis on CCTA with myocardial ischemia on PET (absolute stress myocardial perfusion ≤ 2.4 mL/g/min in ≥ 1 segment). Three models were developed to predict obstructive CAD-induced myocardial ischemia using logistic regression analysis: (1) basic model: including age, sex and cardiac symptoms, (2) risk factor model: adding number of risk factors to the basic model, and (3) CACS model: adding CACS to the risk factor model. Model performance was evaluated using discriminatory ability with area under the receiver-operating characteristic curves (AUC). A total of 647 patients (mean age 62 ± 9 years, 45% men) underwent CACS and sequential CCTA and PET myocardial perfusion imaging. Obstructive CAD with myocardial ischemia on PET was present in 151 (23%) patients. CACS was independently associated with myocardial ischemia (*P* < .001). AUC for the discrimination of ischemia for the CACS model was superior over the basic model and risk factor model (*P* < .001).

**Conclusions:**

Adding CACS to the model including age, sex, cardiac symptoms and number of risk factors increases the accuracy to predict obstructive CAD with myocardial ischemia on PET in symptomatic patients with suspected CAD.

**Supplementary Information:**

The online version contains supplementary material available at 10.1007/s12350-022-03132-z.

## Introduction

Traditionally, myocardial ischemia has been the gatekeeper for invasive coronary angiography and subsequent revascularization.^1^ However, many symptomatic patients with suspected coronary artery disease (CAD) do not have myocardial ischemia.^2–5^ Hence, alternative strategies are warranted in order to improve the referral for ischemia testing of this specific group of patients. Currently, European guidelines recommend physicians to estimate the pre-test probability of obstructive CAD—as a surrogate of myocardial ischemia– using the Diamond-Forrester approach by integrating age, sex and cardiac symptoms.^6,7^ Additional information on the clinical profile of patients, such as the presence and extent of risk factors for cardiovascular disease and coronary artery calcium (CAC), holds potential to further refine these often overestimating pre-test probabilities of myocardial ischemia.^7,8^ Coronary artery calcium scoring (CACS) seems particularly desirable since it is easily performed using non-contrast computed tomography (CT), requiring no intravenous contrast, low radiation exposure and lower costs (as compared to contrast-enhanced CT).^9^ Also, the extent of CACS has been described to correlate well with ischemia.^10,11^ Nevertheless, the optimal use of CACS in improving the pre-test probability assessment of ischemia has yet to be established in a large contemporary patient cohort.^7^ Therefore, the present study aimed to compare three models to predict obstructive CAD with myocardial ischemia on positron emission tomography (PET) in symptomatic patients with suspected CAD: (1) a basic model: including age, sex and cardiac symptoms, (2) a risk factor model: adding number of risk factors to the basic model, and (3) a CACS model: adding CACS to the risk factor model.

## Methods

### Study design and patients

**Figure 1:**
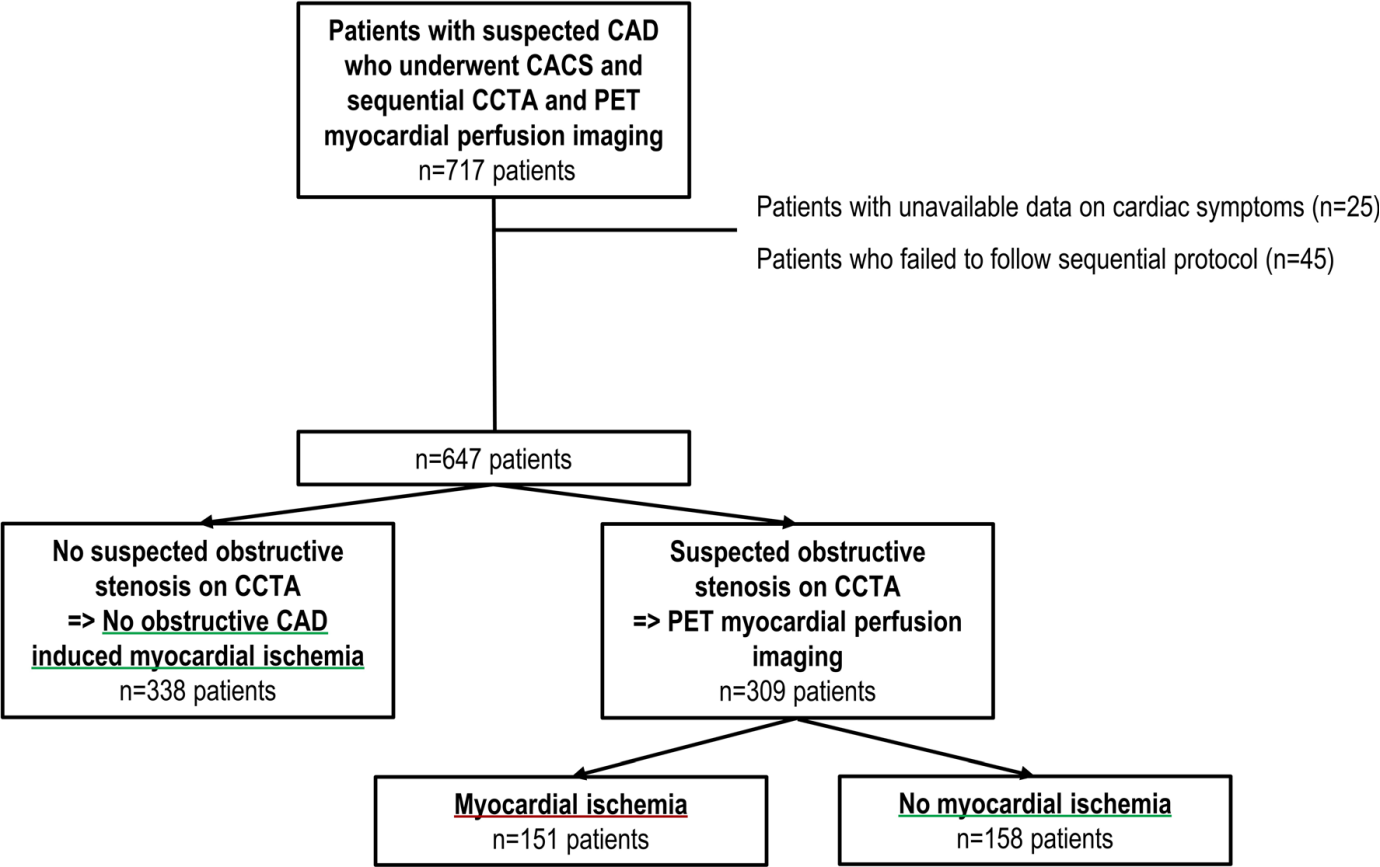
Flow chart of study population. *CACS*, coronary artery calcium score; *CAD*, coronary artery disease; *CCTA*, coronary computed tomography angiography; *PET*, positron emission tomography

The study population included consecutive symptomatic patients with suspected CAD, who were referred for a PET/CT evaluation at the Turku University Hospital, Turku, Finland between 2007 and 2011. A detailed study design has been previously published.^12^ Of those enrolled, 717 patients underwent (1) CACS and (2) sequential coronary computed tomography angiography (CCTA) and [^15^O]H_2_O PET myocardial perfusion imaging to detect potential myocardial ischemia. The ethics committee of the Hospital District of South-West Finland approved the study protocol and waived the need for patients’ written informed consent. The study complied with the principles of the Declaration of Helsinki. Patients with unavailable data on cardiac symptoms (n = 25) or who failed to follow the sequential protocol (n = 45) were excluded. Hence, the present study consisted of 647 patients (Figure 1).

### Image acquisition and analysis

Patients were scanned using a hybrid 64-detector row PET/CT scanner (GE Discovery VCT or GE D690, General Electric Medical Systems, Waukesha, Wisconsin). Protocols regarding image acquisition and analysis have been reported in detail.^12,13^

#### CACS

CACS was calculated from non-contrast CT scans according to the Agatston algorithm.^14^ Scores were categorized into 0, 1–99, 100–399 and ≥ 400.

#### Sequential CCTA and PET myocardial perfusion imaging

CCTA was performed using intravenous low-osmolar iodine (48–155 mL; 320–400 mg/mL) as a contrast agent.^12,13^ Prior to acquisition, intravenous metoprolol (0–30 mg) was administered to achieve heart rates < 60/min. Sublingual nitroglycerin (800 μg) or isosorbide dinitrate (1.25 mg) was administered to achieve maximal coronary vasodilatation. Subsequently, according to study design, all patients with a suspected obstructive stenosis ≥ 50% on CCTA by visual inspection of the attending physician underwent PET myocardial perfusion imaging to detect potential myocardial ischemia. PET myocardial perfusion imaging was performed using dynamic acquisition with [^15^O]H_2_O as a radiotracer (mean radioactivity: 1042 ± 117 MBq).^12,13^ At rest, [^15^O]H_2_O (Radiowater Generator, Hidex Oy, Finland) was intravenously injected over 15 s.^13^ For stress, adenosine (rate: 140 µg/kg/min) was infused 2 min before the stress scan to induce maximal vasodilation. Patients received instructions to avoid caffeine 24 h prior to the scan, considering its interaction with adenosine. Stress scans were quantitatively analyzed according to the 17-segment American Heart Association model using dedicated software (Carimas version 1.1.0, Turku, Finland) by an experienced physician, blinded to clinical or other data.^15,16^ Absolute stress myocardial perfusion was generated in mL/g/min for the segments and left ventricle as a whole (not for all).

### Obstructive CAD-induced myocardial ischemia

The reference standard for myocardial ischemia was defined as an absolute stress myocardial perfusion ≤ 2.4 mL/g/min in ≥ 1 segment on PET.^12^ PET myocardial perfusion imaging was not performed in patients without a suspected obstructive stenosis on CCTA by study design. This specific group was considered to not have obstructive CAD-induced myocardial ischemia.

### Statistical analysis

Normally and non-normally distributed continuous data are presented as means ± standard deviations (SD) and medians with interquartile ranges (IQR), respectively. Categorical data are presented as frequencies with percentages. First, comparisons of continuous data were performed with the Independent-Samples *T* test, Mann–Whitney *U* test, one-way analysis of variance or Kruskal–Wallis test, as appropriate. Comparisons of categorical data were performed using the *χ*^2^ test. Also, the Diamond-Forrest approach was applied to visualize the distribution of obstructive CAD with myocardial ischemia among patients according to age, sex and cardiac symptoms.^6,7^ Additionally, negative predictive values (NPV) and positive predictive values (PPV) were calculated with different cut-points of CACS. Second, models were developed for the prediction of obstructive CAD-induced myocardial ischemia using logistic regression analysis. Uni- and multivariate logistic regression analysis was performed to assess the association between selected variables versus myocardial ischemia. In a stepwise manner, three prediction models were defined: (1) basic model: including age, sex and cardiac symptoms, (2) risk factor model: adding number of risk factors to the basic model, and (3) CACS model: adding CACS to the risk factor model. Measures of association were expressed as odds ratios (OR) with 95% confidence intervals (CI). Goodness of model fit was compared with the likelihood ratio test. Third, performance of the models was evaluated using discriminatory ability. Discriminatory ability was assessed using area under the receiver-operating characteristic curves (AUC), integrated discrimination improvement (IDI) and net reclassification improvement (NRI). AUCs were compared with the DeLong's test.^17,18^ A two-sided *P*-value of < .05 was considered statistically significant, and all statistical analyses were performed with R (version 3.0.3, R Development Core Team, Vienna, Austria), SPSS software (version 26, SPSS IBM Corp., Armonk, New York) and MedCalc software (version 19.2.0, Ostend, Belgium).

## Results

### Patients

Baseline characteristics of the patients are shown in Table 1. In total, 647 patients (mean age 62 ± 9 years, 45% men) underwent CACS and sequential CCTA and [^15^O]H_2_O PET myocardial perfusion imaging for ischemia assessment. CCTA ruled out an obstructive stenosis in 338 patients; they were considered to not have obstructive CAD-induced myocardial ischemia (and did not undergo PET myocardial perfusion imaging by the sequential study design) (Figure 1). CCTA revealed a suspected obstructive stenosis in 309 patients. Obstructive CAD with myocardial ischemia on PET was present in 151 (23% out of 647) patients. Patients with myocardial ischemia were older (63 ± 8 years vs. 61 ± 10 years, *P* = .002), more often male (72% vs. 37%, *P* < .001) and presented more frequently with typical angina (37% vs. 22%, *P* < .001) as compared to patients without ischemia. In addition, patients with myocardial ischemia had more risk factors for cardiovascular disease (*P* < .001) and used more medications (*P* ≤ .007). The distribution of ischemia among patients based on the Diamond-Forrester approach according to age, sex and cardiac symptoms was demonstrated in Supplemental Table 1.

**Table 1:** Baseline characteristics according to the presence of obstructive CAD-induced myocardial ischemia

	Total cohort n = 647	Ischemia n = 151	No ischemia n = 496	*P*-value
Age, years	62 ± 9	63 ± 8	61 ± 10	**.002**
Male	294 (45)	109 (72)	185 (37)	**< .001**
BMI, kg/m^2^	28.1 ± 4.8	28.7 ± 4.9	27.7 ± 4.6	**.038**
*Cardiac symptoms*				
Non-anginal pain	59 (9)	8 (5)	51 (10)	.062
Atypical angina	256 (40)	48 (32)	208 (42)	**.026**
Typical angina	163 (25)	56 (37)	107 (22)	**< .001**
Dyspnea at exertion	169 (26)	39 (26)	130 (26)	.925
*Cardiac risk factors*				
Hypertension	459 (71)	128 (85)	331 (67)	**< .001**
Dyslipidemia	412 (64)	118 (78)	294 (59)	**< .001**
Diabetes mellitus	92 (14)	35 (23)	57 (12)	**< .001**
Family history of CAD	289 (45)	69 (46)	220 (44)	.772
Smoking current or former	227 (35)	72 (48)	155 (31)	**< .001**
Number of risk factors*	2 ± 1	3 ± 1	2 ± 1	**< .001**
*Cardiac medication*				
Aspirin	356 (65)	108 (83)	248 (59)	**< .001**
Beta blockers	309 (56)	95 (71)	214 (51)	**< .001**
Calcium channel blockers	85 (16)	23 (18)	62 (15)	.489
Renin-angiotensin system inhibitors	218 (40)	67 (49)	151 (36)	**.007**
Statins	285 (52)	93 (69)	192 (46)	**< .001**
*Laboratory findings*				
Total cholesterol, mmol/l	4.9 ± 1.0	4.9 ± 1.1	4.9 ± .9	.781
Low-density lipoprotein, mmol/L	2.7 ± .9	2.8 ± 1.0	2.7 ± .8	.495
High-density lipoprotein, mmol/L	1.6 ± .5	1.4 ± .4	1.6 ± .5	**< .001**
Triglycerides, mmol/L	1.5 ± 1.0	1.8 ± 1.2	1.4 ± .9	**.009**
Creatinine, µmol/L	75.8 ± 15.1	81.4 ± 15.5	74.1 ± 14.5	**< .001**

Bold values are statistically significant (*P* < .05)

Values are presented as mean ± SD or n (%)

*BMI*, body mass index; *CAD*, coronary artery disease. Definitions: *Including hypertension, dyslipidemia, diabetes mellitus, family history of CAD and smoking current or former

### Imaging findings

#### CACS

**Figure 2:**
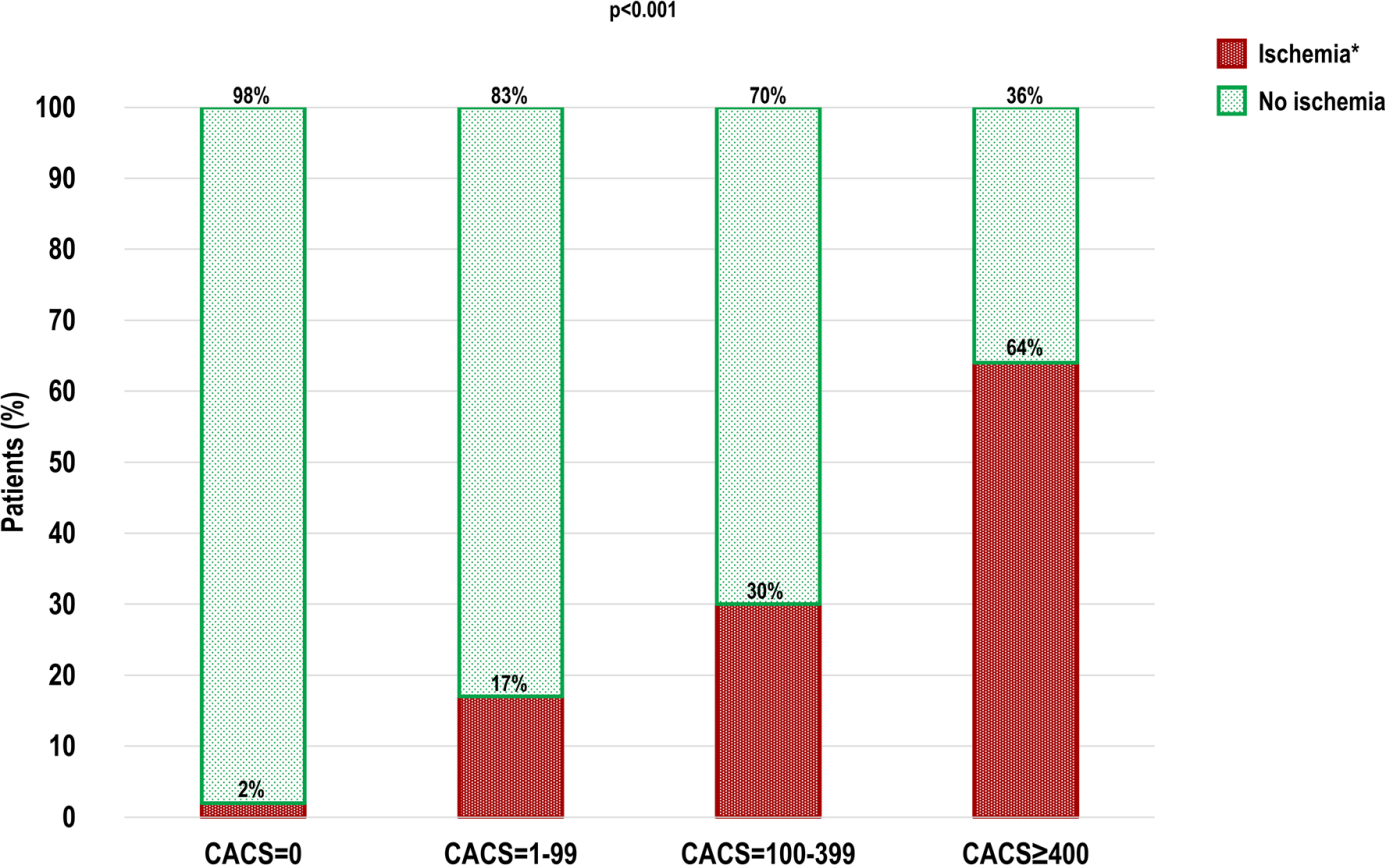
Bar graphs of obstructive CAD-induced myocardial ischemia by CACS. *CACS*, coronary artery calcium scoring; *CAD*, coronary artery disease; *PET*, positron emission tomography. Definitions: *In patients with obstructive CAD-induced myocardial ischemia, a median of 5 segments (IQR 1–13 segments) for CACS = 0, 6 segments (IQR 3–12 segments) for CACS = 1–99, 7 segments (IQR 4–12 segments) for CACS = 100–399 and 12 segments (IQR 6–16 segments) for CACS ≥ 400 was affected

**Figure 3:**
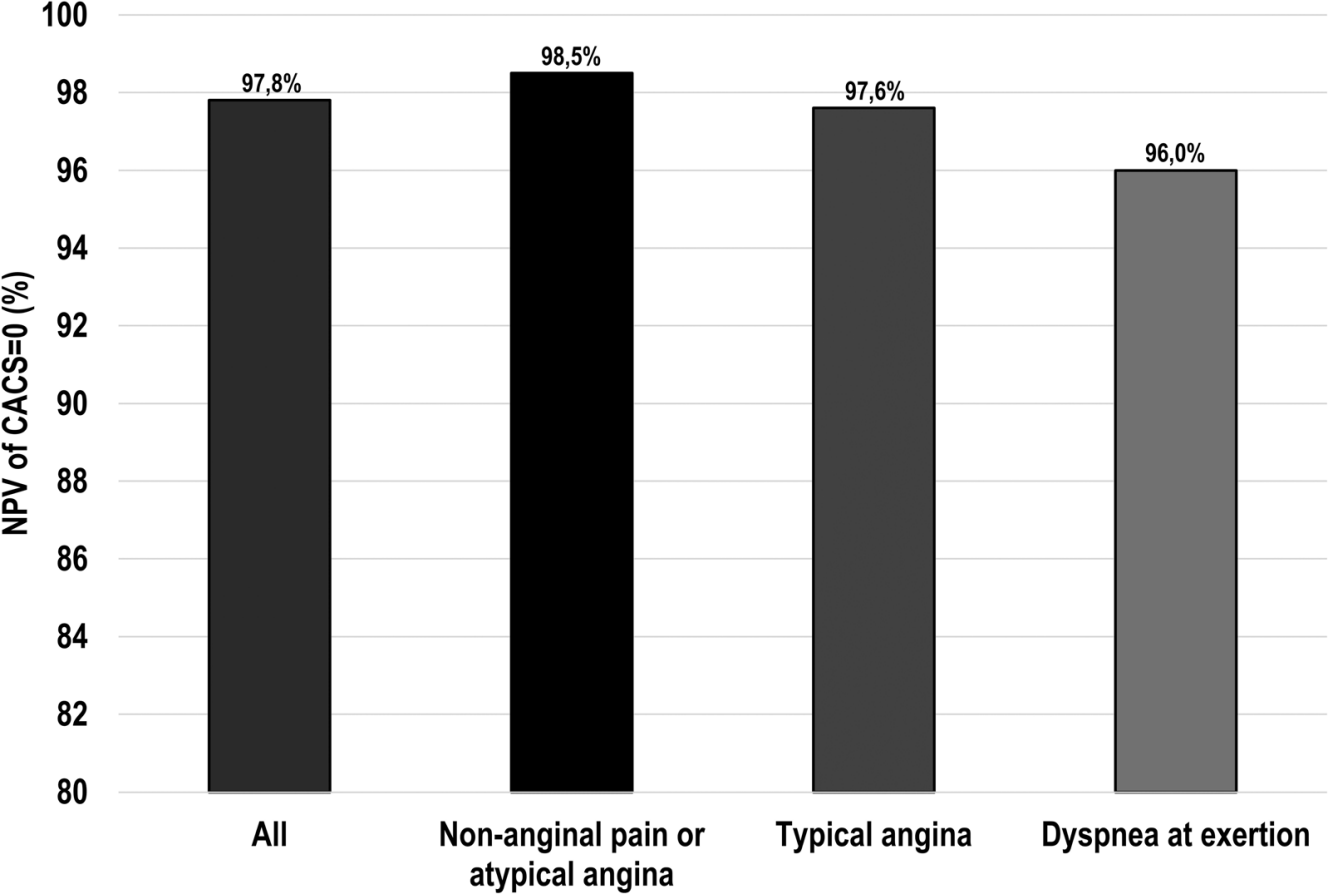
Bar graphs of NPV of CACS = 0 by cardiac symptoms. *CACS*, coronary artery calcium scoring; *NPV*, negative predictive value

Median CACS of the patients was 32 (IQR 0–281) (Table 2). In total, 225 (35%) and 422 (65%) patients had CACS = 0 and CACS ≥ 1, respectively. Patients with obstructive CAD-induced myocardial ischemia had a higher CACS as compared to patients without ischemia (422 (IQR 117–1047) vs. 5 (IQR 0–136), *P* < .001). The majority of patients with ischemia had CACS ≥ 400 (53%). Moreover, the frequency of ischemia increased with higher CACS categories: 2% for CACS = 0, 17% for CACS = 1–99, 30% for CACS = 100–399 and 64% for CACS ≥ 400 (*P* < .001) (Figure 2). Consequently, the NPV of CACS = 0 was 97.8% (95% CI 94.9–99.1%) and this value slightly varied according to the cardiac symptoms at presentation: 98.5% for patients with non-anginal pain or atypical angina, 97.6% for patients with typical angina and 96.0% for patients with dyspnea at exertion (Fig. 3). Conversely, the PPV of CACS ≥ 1 was only 34.6% (95% CI 32.7–36.5%) and also differed according to symptomatic status: 29.7% for patients with non-anginal or atypical angina, 45.5% for patients with typical angina and 31.1% for patients with dyspnea at exertion. When the cut-point was set at CACS < 100 versus CACS ≥ 100, NPV and PPV were 91.6% (95% CI 88.9–93.7%) and 46.5% (95% CI 42.4–50.6%), respectively.

**Table 2:** CACS findings according to the presence of obstructive CAD-induced myocardial ischemia

	Total cohort n = 647	Ischemia n = 151	No ischemia n = 496	*P*-value
*CACS*	32 (0–281)	422 (117–1047)	5 (0–136)	**< .001**
0	225 (35)	5 (3)	220 (44)	**< .001**
1–99	168 (26)	28 (19)	140 (28)	**.017**
100–399	129 (20)	38 (25)	91 (18)	.066
≥ 400	125 (19)	80 (53)	45 (9)	**< .001**
*PET myocardial perfusion imaging*				
Global stress myocardial perfusion, mL/g/min	–	2.3 ± .7	3.9 ± .9*	**< .001**

Bold values are statistically significant (*P* < .05)

Values are presented as mean ± SD, median (IQR) or n (%)

*CACS*, coronary artery calcium score; *CAD*, coronary artery disease; *PET*, positron emission tomography. Definitions: *Values only available for patients who underwent PET myocardial perfusion imaging, as depicted in Figure 1

#### Sequential CCTA and PET myocardial perfusion imaging

Details regarding sequential CCTA and PET myocardial perfusion imaging are shown in Figure 1. In patients with obstructive CAD-induced myocardial ischemia, a median of 10 segments (IQR 5–15 segments) was affected. Patients with myocardial ischemia had a reduced global stress myocardial perfusion as compared to patients without ischemia on PET (2.3 ± .7 mL/g/min vs. 3.9 ± .9 mL/g/min, *P* < .001) (Table 2).

### Prediction of obstructive CAD with myocardial ischemia

#### Model development using logistic regression analysis

In the univariable analysis, age, male sex, typical angina, all individual cardiac risk factors (except for family history of CAD) and the number of risk factors per-patient were each associated with obstructive CAD-induced myocardial ischemia (*P* ≤ .005). Furthermore, CACS was a significant univariable predictor of myocardial ischemia, both as a continuous (*P* < .001) and categorized score (*P* < .001) (Table 3). In the multivariable analysis, prediction models of ischemia were defined using a stepwise approach: (1) basic model: including age, sex and cardiac symptoms, (2) risk factor model: adding number of risk factors to the basic model, and (3) CACS model: adding CACS to the risk factor model (Table 4). In the CACS model, male sex (OR 4.686 (95% CI 2.921–7.518), *P* < .001), typical angina (OR 4.555 (1.636–12.682), *P* = .004), dyspnea at exertion (OR 3.026 (95% CI 1.078–8.495), number of risk factors (OR 1.461 (95% CI 1.191–1.793), *P* < .001) and CACS (OR 1.002 (95% CI 1.001–1.002), *P* < .001) remained independently associated with myocardial ischemia. Importantly, adding CACS to the risk factor model resulted in a significantly better fit of the model (*χ*^2^ = 200 vs. *χ*^2^ = 126, *P* < .001).

**Table 3:** Univariate association between clinical profile, CACS and obstructive CAD-induced myocardial ischemia

	OR (95% CI)	*P*-value
Age	1.030 (1.009–1.051)	**.005**
Male	4.363 (2.925–6.507)	**< .001**
*Cardiac symptoms*		
Non-anginal pain	Ref	**—**
Atypical angina	1.471 (.655–3.303)	.349
Typical angina	3.336 (1.481–7.518)	**.004**
Dyspnea at exertion	1.912 (.837–4.372)	.124
*Cardiac risk factors*		
Hypertension	2.774 (1.714–4.490)	**< .001**
Dyslipidemia	2.457 (1.605–3.760)	** < .001**
Diabetes mellitus	2.324 (1.455–3.711)	** < .001**
Family history of CAD	1.056 (.732–1.522)	.772
Smoking current or former	2.005 (1.383–2.907)	** < .001**
Number of risk factors*	1.694 (1.425- 2.013)	** < .001**
*CACS*	1.002 (1.002–1.003)	** < .001**

Bold values are statistically significant (*P* < .05)

*CACS*, coronary artery calcium score; *CAD*, coronary artery disease. Definitions: *Including hypertension, dyslipidemia, diabetes mellitus, family history of CAD and smoking current or former

**Table 4:** Multivariate association between clinical profile, CACS and obstructive CAD-induced myocardial ischemia

	Basic model		Risk factor model		CACS model	
	OR (95% CI)	*P*-value	OR (95% CI)	*P*-value	OR (95% CI)	*P*-value
*Age*	1.036 (1.013–1.060)	**.002**	1.039 (1.014–1.064)	**.002**	1.009 (.982–1.036)	.519
*Male*	5.347 (3.511–8.142)	**< .001**	5.593 (3.623–8.635)	**< .001**	4.686 (2.921–7.518)	**< .001**
*Cardiac symptoms*						
Non-anginal pain	Ref	**—**	Ref	—	Ref	**—**
Atypical angina	1.796 (.779–4.139)	.169	1.486 (.630–3.507)	.366	2.105 (.770–5.756)	.147
Typical angina	4.155 (1.772–9.742)	**.001**	3.336 (1.391–8.000)	**.007**	4.555 (1.636–12.682)	**.004**
Dyspnea at exertion	2.364 (.995–5.613)	.051	1.935 (.800–4.679)	.143	3.026 (1.078–8.495)	**.035**
Number of risk factors*			1.723 (1.428–2.079)	**< .001**	1.461 (1.191–1.793)	**< .001**
*CACS*					1.002 (1.001–1.002)	**< .001**

Bold values are statistically significant (*P* < .05)

*CACS*, coronary artery calcium score; *CAD*, coronary artery disease. Definitions: *Including hypertension, dyslipidemia, diabetes mellitus, family history of CAD and smoking current or former; ∫Compared with the basic model; †Compared with the risk factor model

#### Model performance using discriminatory ability

**Figure 4:**
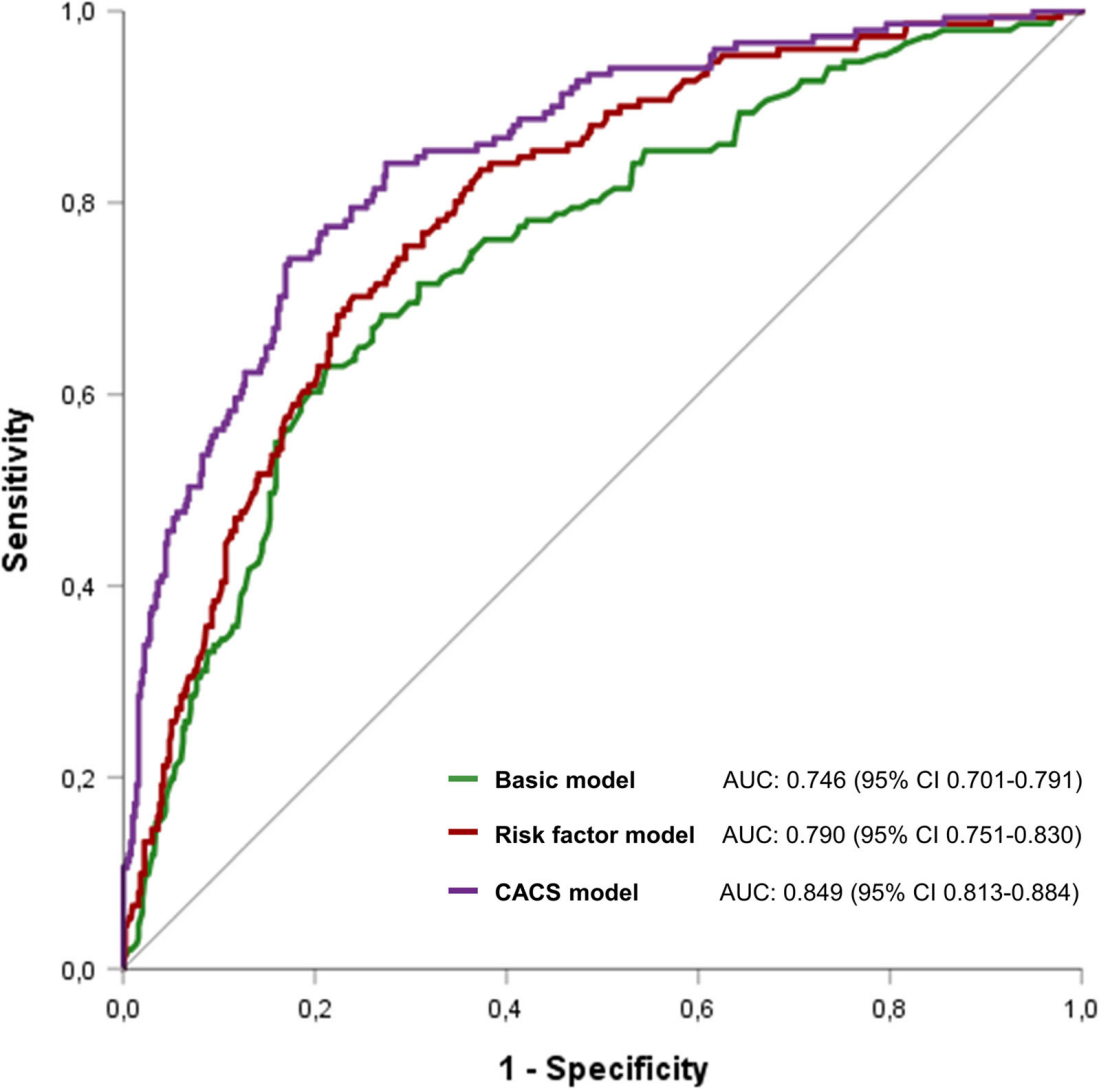
Discriminatory ability of the three models. *AUC*, area under the receiver-operating characteristics curve; *CACS*, coronary artery calcium scoring

AUC for the discrimination of obstructive CAD with myocardial ischemia was .746 (95% CI .701–.791) for the basic model, .790 (95% CI .751–.830) for the risk factor model and .849 (95% CI .813–.884) for the CACS model (Figure 4). The CACS model had a significantly better discriminatory ability than the basic model (*P* < .001) and risk factor model (*P* < .001). Also, the CACS model provided incremental predictive information over the basic model (IDI = .176, *P* < .001 and NRI = .633, *P* < .001) and risk factor model (IDI = .125, *P* < .001 and NRI = .440, *P* < .001) (Supplemental Table 2).

## Discussion

The present study evaluated 647 symptomatic patients with suspected CAD from a large contemporary patient cohort, who underwent CACS and sequential CCTA and [^15^O]H_2_O PET myocardial perfusion imaging for ischemia assessment. We compared three models to predict obstructive CAD with myocardial ischemia on PET: (1) a basic model, (2) a risk factor model and (3) a CACS model. CACS was strongly and independently associated with myocardial ischemia. Moreover, by incorporating CACS into the pre-test probability assessment, the discrimination of ischemia significantly improved compared to the basic model and risk factor model. These findings suggest a possible role for routine CACS detection in symptomatic patients in order to refine referral for ischemia testing, by either triaging them away from (in case of low CACS) or towards (in case of high CACS) this test. Particularly, the NPV of CACS = 0 was excellent (97.8%) irrespective of the cardiac symptoms at presentation (96.0–98.5%). Our approach is an example of the stepwise application of non-invasive imaging tests, which in turn could lead to more cost-effective care.

### CACS in asymptomatic patients: preventative care

Anatomical imaging with CACS has been initially introduced as a screening tool for CAD in asymptomatic patients with the aim of improving cardiovascular risk assessment and guiding primary preventative care.^19–21^ Regarding cardiovascular risk assessment, various large long-term population-based studies have uniformly reported on the association between CACS and major adverse cardiac events in asymptomatic patients without known CAD.^22–25^ Especially, a CACS = 0 has been linked to a very low risk of adverse events (power of zero).^23,26,27^ Regarding preventative care strategies, it has been clearly demonstrated that a CACS = 0 can reclassify a large subset of asymptomatic patients (44%) in whom statins would have been otherwise considered or recommended (atherosclerotic cardiovascular disease risk score ≥ 5%) according to existing guidelines.^28^

### CACS in symptomatic patients: ischemia

On the other hand, anatomical imaging in symptomatic patients with suspected CAD has the aim to identify hemodynamically obstructive CAD (coronary artery stenosis ≥ 50%) that causes ischemia.^29^ Few studies have reported on the association between CACS and myocardial ischemia on PET in symptomatic patients with suspected CAD.^30–32^ Schenker et al. analyzed 695 symptomatic patients with suspected CAD, who underwent CACS and PET myocardial perfusing imaging using a hybrid PET/CT scanner.^30^ In line with our results, a stepwise increase was demonstrated in the frequency of myocardial ischemia with increasing CACS (16% for CACS = 0 to 49% for CACS ≥ 1000). Furthermore, adding CACS to a model including age, sex, cardiac symptoms and risk factors improved the discrimination of ischemia significantly (AUC .72 vs. AUC .67, *P* < .001). Likewise, Esteves et al. evaluated 84 symptomatic patients with a low-intermediate likelihood of CAD, who were admitted to the chest pain unit and underwent CACS plus myocardial ischemia testing with PET.^31^ Applying this strategy, a strong association was shown between CACS = 0 and the absence of myocardial ischemia, yielding a negative predictive value of 100%. Again, these results were overall highly consistent with the findings in the current study, showing only a 2% prevalence of myocardial ischemia in patients with CACS = 0. Similar findings were derived from studies using single photon emission computed tomography as the reference standard for myocardial ischemia.^11,29^ However, it should be noted that PET has enhanced diagnostic performance over single photon emission computed tomography, in particular when myocardial perfusion is quantitatively analyzed.^33^ Additionally, all latest generation PET scanners are combined with a CT scanner into a hybrid system, of which the low-dose non-gated CT transmission scan can be used to not only perform attenuation correction of the PET images but also to perform visual assessment of CAC.^34,35^ With the rapid development of artificial intelligence with sophisticated algorithms, this approach holds potential for the automated assessment of CAC from non-gated CT scans.^36,37^

### Limitations

Some limitations of the present study need to be addressed. First, our study had a retrospective observational design with limitations such as (unmeasured) confounding factors and selection bias. For instance, of those enrolled in the registry, CACS was performed per protocol in all patients for risk stratification purposes, but not analyzed in some patients due to logistical or technical reasons.^12^ Second, PET myocardial perfusion imaging was not performed in patients without suspected obstructive stenosis on CCTA according to study design. Absence of myocardial ischemia in this specific group of patients was therefore assumption-based, but in line with published literature.^31^ Nevertheless, we acknowledge that diffuse, heterogenous CAD or microvascular dysfunction could have contributed to downstream myocardial perfusion abnormalities.^38–41^ Unfortunately, we were not able to analyze this in detail due to the sequential design of the study. Third, PET myocardial perfusion findings were solely interpreted on a per-patient basis, since CACS was not available on a per-vessel basis. Lastly, utilizing CACS as a gatekeeper to ischemia testing still needs prospective and randomized data. However, our study adds to the wealth of data suggesting that patients with CACS = 0 are at low risk (but not risk free). To this end it should be emphasized that clinical decisions should always be individualized.

## New knowledge gained

The stepwise application of non-invasive imaging tests, including an initial CACS, can potentially refine the referral for ischemia testing of symptomatic patients with CAD.

## Conclusion

In symptomatic patients with suspected CAD, a CACS model including age, sex, cardiac symptoms, number of risk factors and CACS allows for accurate and superior prediction of obstructive CAD with myocardial ischemia on PET.

## Supplementary Information

Below is the link to the electronic supplementary material.

Supplementary file1 (DOCX 80 kb)

Supplementary file2 (PPTX 378 kb)

Supplementary file3 (MP3 4127 kb)

## Abbreviations

AUC: Area under the receiver-operating characteristic curve
CAC: Coronary artery calcium
CACS: Coronary artery calcium scoring
CAD: Coronary artery disease
CCTA: Coronary computed tomography angiography
CT: Computed tomography
IDI: Integrated discrimination improvement
NPV: Negative predictive value
NRI: Net reclassification improvement
PET: Positron emission tomography
PPV: Positive predictive value
