# Supplementary material for: Incorporating coronary artery calcium scoring in the prediction of obstructive coronary artery disease with myocardial ischemia: a study with sequential use of coronary computed tomography angiography and positron emission tomography imaging
Source: J Nucl Cardiol. 2022 Nov 15;30(1):178–88. doi: 10.1007/s12350-022-03132-z (PMC9984337; doi:10.1007/s12350-022-03132-z)
Supplement: Supplementary file 1 — Supplementary file1 (DOCX 80 kb) [file 12350_2022_3132_MOESM1_ESM.docx]

**APPENDIX**

**Supplemental Table 1: Obstructive CAD with myocardial ischemia in study population.**

Observed obstructive CAD with myocardial ischemia amongst patients based on the Diamond-Forrester approach according to age, sex and cardiac symptoms. Grey: 0-5% observed ischemia. Light-green: 5-15% observed ischemia. Dark-green: >15% observed ischemia.

Abbreviations: CAD, coronary artery disease.


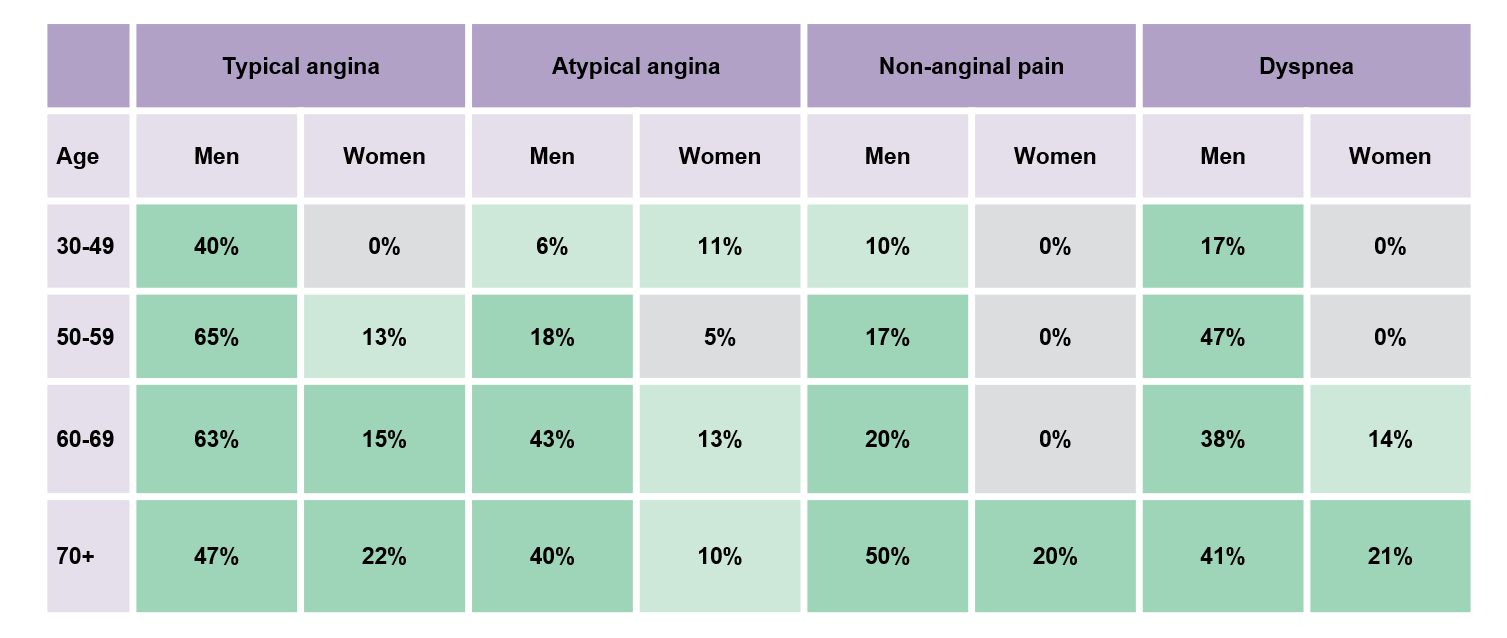


**Supplemental Table 2: Comparison of model performance.**

Abbreviations: AUC, area under the receiver-operating characteristics curve; CACS, coronary artery calcium scoring; IDI, integrated discrimination improvement; NRI, net reclassification improvement.

Definitions: *Compared with the basic model; †Compared with the risk factor model.

|  | **Basic model** | **Risk factor model** | **CACS model** | **p-value** |
| --- | --- | --- | --- | --- |
| **Discriminatory ability** |  |  |  |  |
| IDI (95% CI) | Ref. | 0.051 (0.032-0.071) | 0.176 (0.135-0.218) | **<0.001*** |
|  | - | Ref. | 0.125 (0.088-0.163) | **<0.001**† |
| NRI (95% CI) | Ref. | 0.309 (0.129-0.540) | 0.633 (0.448-0.841) | **<0.001*** |
|  | - | Ref. | 0.440 (0.236-0.656) | **<0.001**† |
