# Supplementary material for: Incorporating coronary artery calcium scoring in the prediction of obstructive coronary artery disease with myocardial ischemia: a study with sequential use of coronary computed tomography angiography and positron emission tomography imaging
Source: J Nucl Cardiol. 2022 Nov 15;30(1):178–88. doi: 10.1007/s12350-022-03132-z (PMC9984337; doi:10.1007/s12350-022-03132-z)
Supplement: Supplementary file 2 — Supplementary file2 (PPTX 378 kb) [file 12350_2022_3132_MOESM2_ESM.pptx]

## Slide 1
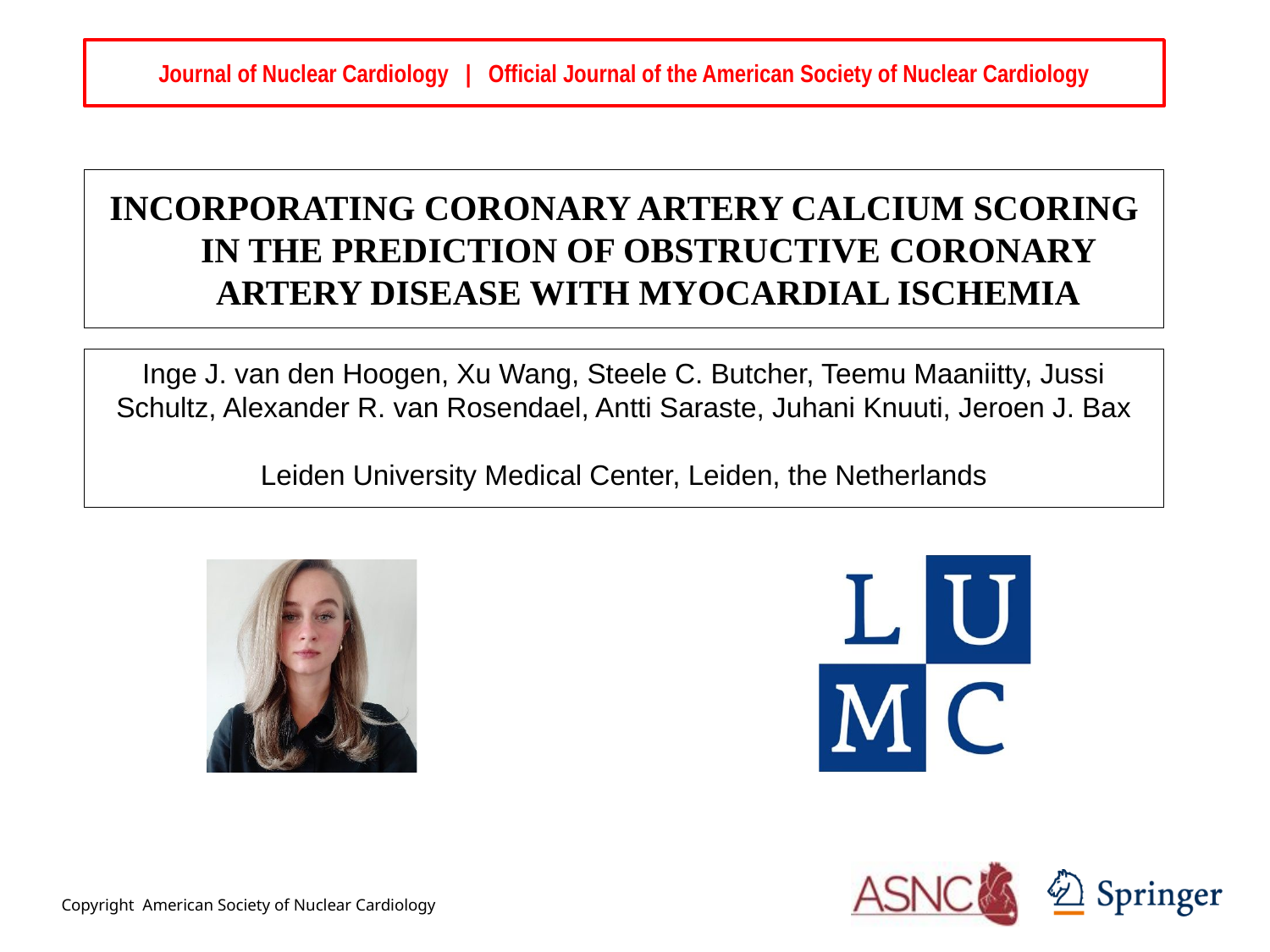

Journal of Nuclear Cardiology | Official Journal of the American Society of Nuclear Cardiology
# Incorporating Coronary Artery Calcium Scoring in the Prediction of Obstructive Coronary Artery Disease with Myocardial Ischemia
Inge J. van den Hoogen, Xu Wang, Steele C. Butcher, Teemu Maaniitty, Jussi Schultz, Alexander R. van Rosendael, Antti Saraste, Juhani Knuuti, Jeroen J. Bax
Leiden University Medical Center, Leiden, the Netherlands
Copyright American Society of Nuclear Cardiology

## Slide 2
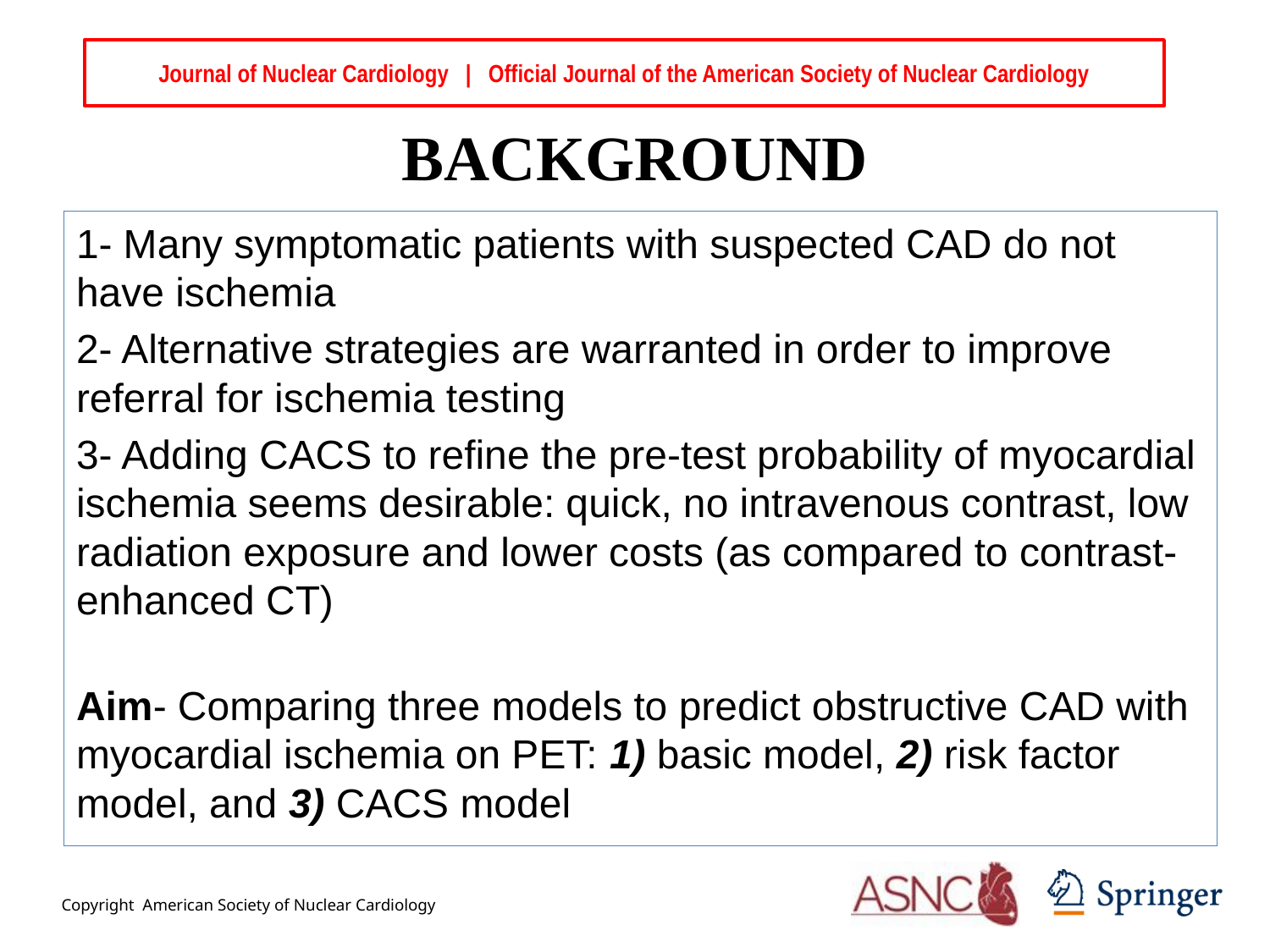

Journal of Nuclear Cardiology | Official Journal of the American Society of Nuclear Cardiology
# BACKGROUND
1- Many symptomatic patients with suspected CAD do not have ischemia
2- Alternative strategies are warranted in order to improve referral for ischemia testing
3- Adding CACS to refine the pre-test probability of myocardial ischemia seems desirable: quick, no intravenous contrast, low radiation exposure and lower costs (as compared to contrast-enhanced CT)
Aim- Comparing three models to predict obstructive CAD with myocardial ischemia on PET: 1) basic model, 2) risk factor model, and 3) CACS model
Copyright American Society of Nuclear Cardiology

## Slide 3
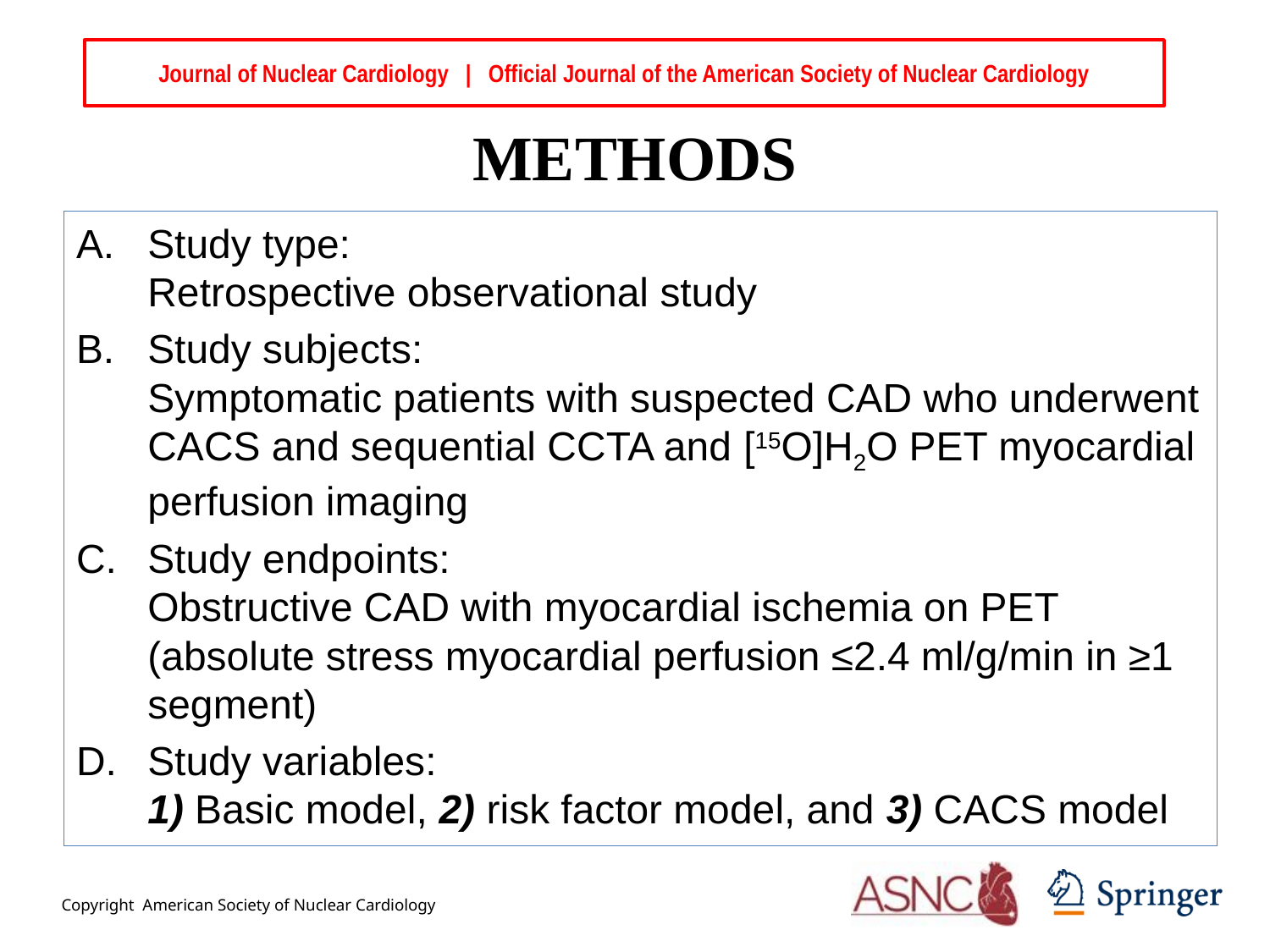

Journal of Nuclear Cardiology | Official Journal of the American Society of Nuclear Cardiology
# METHODS
Study type:
Retrospective observational study
Study subjects:
Symptomatic patients with suspected CAD who underwent CACS and sequential CCTA and [15O]H2O PET myocardial perfusion imaging
Study endpoints:
Obstructive CAD with myocardial ischemia on PET (absolute stress myocardial perfusion ≤2.4 ml/g/min in ≥1 segment)
Study variables:
1) Basic model, 2) risk factor model, and 3) CACS model
Copyright American Society of Nuclear Cardiology

## Slide 4
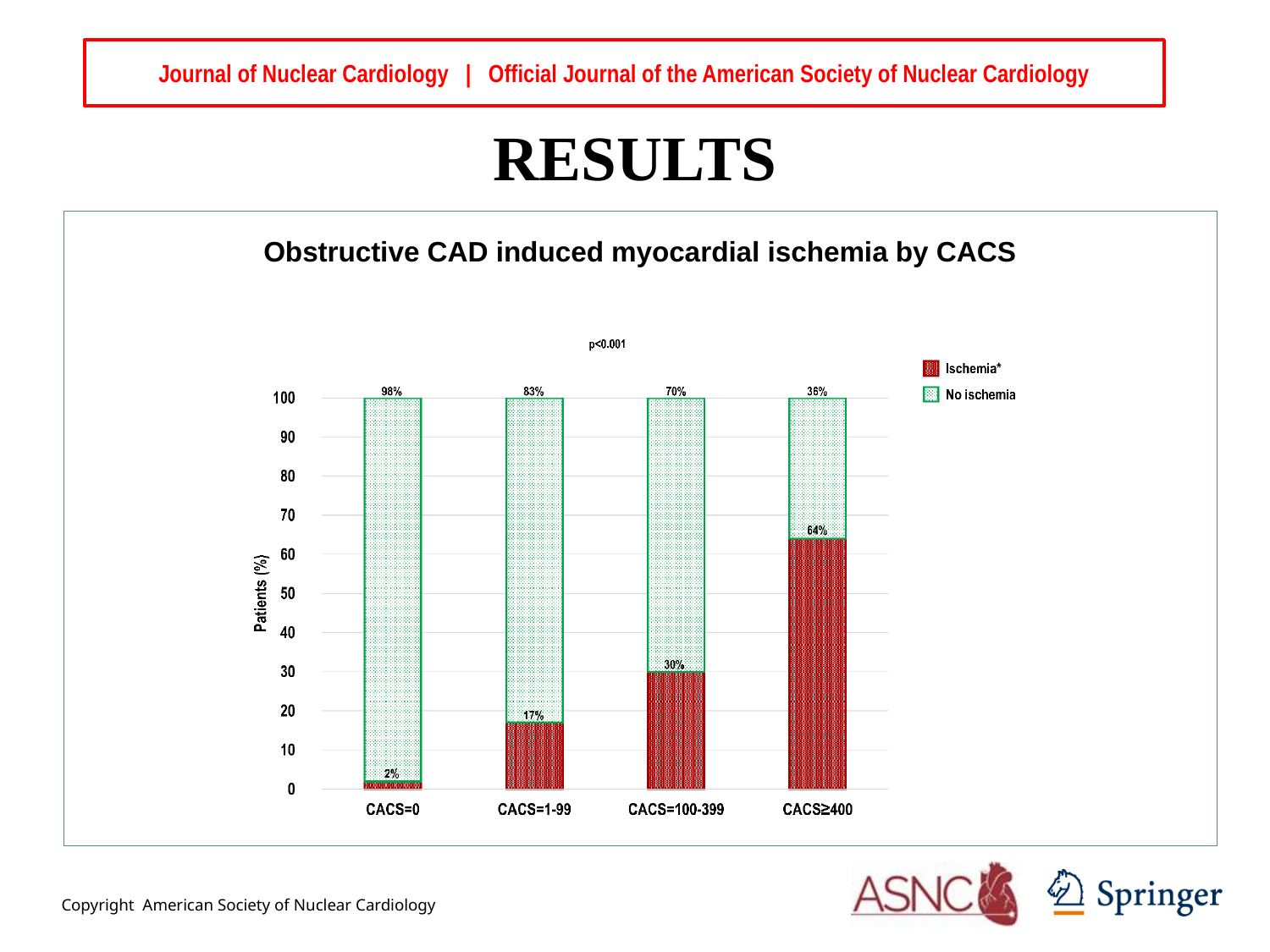

Journal of Nuclear Cardiology | Official Journal of the American Society of Nuclear Cardiology
# RESULTS
Obstructive CAD induced myocardial ischemia by CACS
Copyright American Society of Nuclear Cardiology

## Slide 5
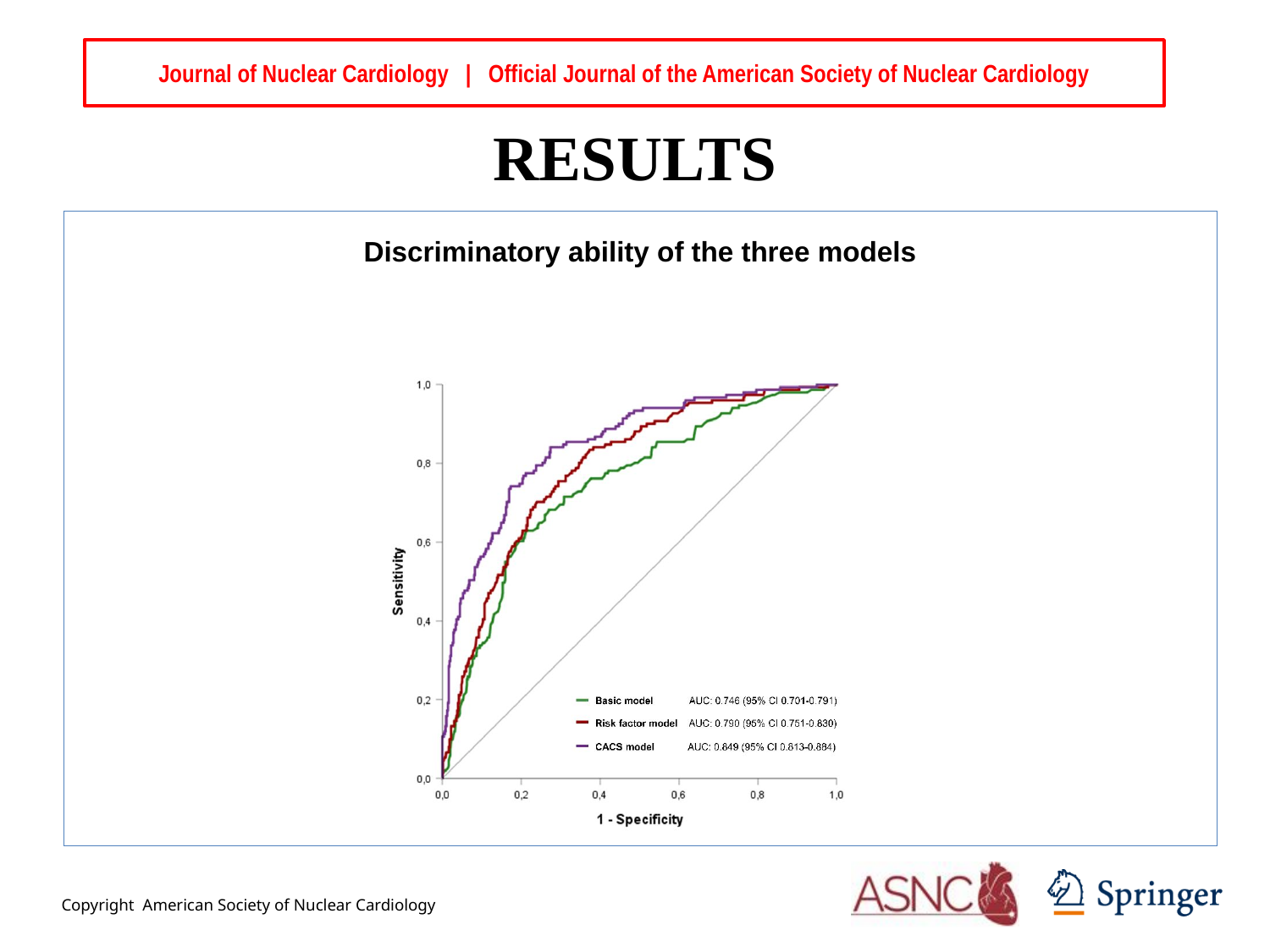

Journal of Nuclear Cardiology | Official Journal of the American Society of Nuclear Cardiology
# RESULTS
Discriminatory ability of the three models
Copyright American Society of Nuclear Cardiology

## Slide 6
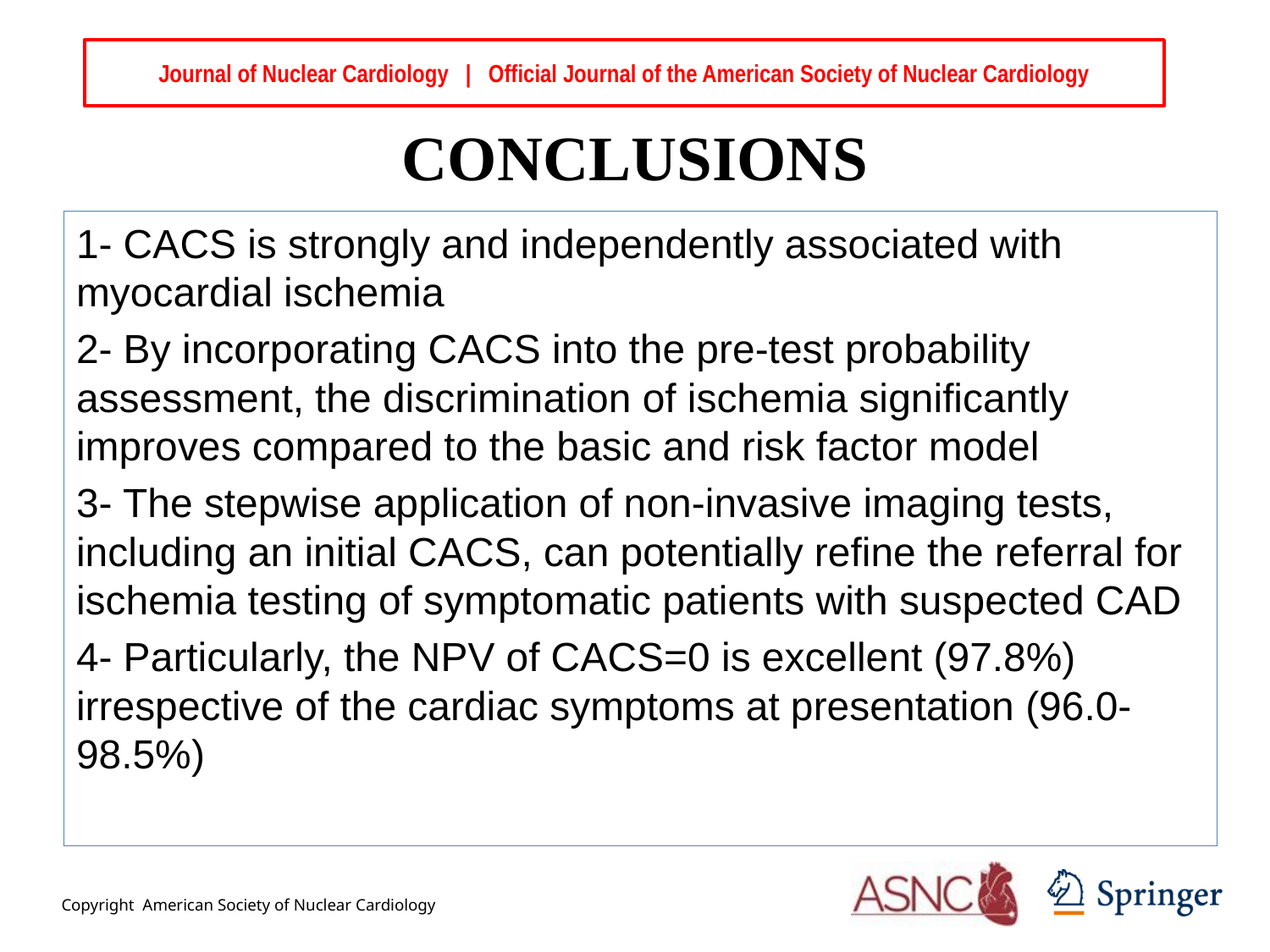

Journal of Nuclear Cardiology | Official Journal of the American Society of Nuclear Cardiology
# CONCLUSIONS
1- CACS is strongly and independently associated with myocardial ischemia
2- By incorporating CACS into the pre-test probability assessment, the discrimination of ischemia significantly improves compared to the basic and risk factor model
3- The stepwise application of non-invasive imaging tests, including an initial CACS, can potentially refine the referral for ischemia testing of symptomatic patients with suspected CAD
4- Particularly, the NPV of CACS=0 is excellent (97.8%) irrespective of the cardiac symptoms at presentation (96.0-98.5%)
Copyright American Society of Nuclear Cardiology
